# Brain Protective Ventilation Strategies in Severe Acute Brain Injury

**DOI:** 10.1007/s11910-025-01462-2

**Published:** 2025-10-13

**Authors:** Sarah Al Sharie, Rahma Almari, Saif Azzam, Lou’i Al-Husinat, Mohammad Araydah, Denise Battaglini, Marcus J. Schultz, Nicolo’ Antonino Patroniti, Patricia RM Rocco, Chiara Robba

**Affiliations:** 1 Division of Allergy, Pulmonary, and Critical Care Medicine, Department of Medicine, Vanderbilt University Medical Center, Nashville, TN United States of America; 2 Faculty of Medicine, Yarmouk University, Irbid, Jordan; 3 Department of Internal Medicine, Istishari Hospital, Amman, Jordan; 4 Department of Surgical Sciences and Integrated Diagnostics (DISC), University of Genova, Genova, Italy; 5 Anesthesia and Intensive Care, IRCCS Ospedale Policlinico San Martino, Genova, Italy; 6 Department of Intensive Care, Amsterdam University Medical Centers, Amsterdam, The Netherlands; 7 Department of Anesthesia, General Intensive Care and Pain Management, Medical University, Wien, Vienna Austria; 8 Nuffield Department of Medicine, University of Oxford, Oxford, United Kingdom; 9 Mahidol-Oxford Research Unit (MORU), Mahidol University, Bangkok, Thailand; 10 Laboratory of Pulmonary Investigation, Carlos Chagas Filho Institute of Biophysics, Federal University of Rio de Janeiro, Rio de Janeiro, Brazil; 11 IRCCS Policlinico San Martino, Genova, 010/5551 Italy

**Keywords:** Mechanical ventilation, Neurocritical care, Intracranial pressure, Cerebral perfusion, Lung-protective ventilation, Brain injury

## Abstract

**Purpose of the review:**

This narrative review synthesizes ten key evidence-based principles for optimizing ventilatory management in patients with acute brain injury, including traumatic brain injury, stroke, and hypoxic-ischemic encephalopathy. Recent findings: We emphasize the need to individualize ventilator settings to preserve intracranial pressure (ICP) and cerebral perfusion pressure (CPP), while maintaining lung-protective strategies. Key recommendations include prioritizing physiological targets over ventilator modes, judicious use of positive end-expiratory pressure (PEEP) with concurrent cerebral monitoring, limiting plateau pressures, and maintaining tidal volumes within protective ranges. Minimizing driving pressure (ΔP) and mechanical power (MP) is emphasized to reduce the risk of ventilator-induced lung injury (VILI). The review underscores the importance of precise control of arterial carbon dioxide (PaCO₂) to regulate cerebral blood flow, avoidance of both hypoxemia and hyperoxia, and the integration of multimodal neuromonitoring to inform ventilatory decisions. Additional considerations include the potential benefits of early tracheostomy in patients requiring prolonged ventilation, as well as the influence of sedation depth, fluid management, and autoregulation monitoring on outcomes.

**Summary:**

By aligning respiratory support with cerebral pathophysiology, clinicians can mitigate secondary brain injury and enhance recovery in this vulnerable population.

## Introduction

Mechanical ventilation (MV) is a cornerstone of supportive care in patients with severe brain injury, ensuring adequate oxygenation, carbon dioxide (CO₂) regulation, and respiratory mechanics when spontaneous breathing is compromised [1]. In neurocritical care, however, MV management extends beyond conventional pulmonary concerns, as ventilatory parameters can significantly influence intracranial pressure (ICP), cerebral perfusion pressure (CPP), and ultimately neurological outcomes [2]. The complex interaction between cerebral and respiratory physiology presents unique challenges in this population [3].

A substantial proportion of neurocritically ill patients—particularly those with traumatic brain injury (TBI), subarachnoid hemorrhage (SAH), or hypoxic–ischemic encephalopathy (HIE)—require mechanical ventilation during their intensive care unit (ICU) stay, especially in the acute phase. More than 60% of such patients necessitate ventilatory support. However, lung-protective ventilation strategies commonly employed in general ICU populations, such as those with acute respiratory distress syndrome (ARDS), may not be directly applicable to brain-injured patients due to distinct physiological concerns. For example, permissive hypercapnia, a component of lung-protective ventilation, can exacerbate intracranial hypertension via cerebral vasodilation [4]. Similarly, while positive end-expiratory pressure (PEEP) improves oxygenation, it may elevate intrathoracic pressure and subsequently increase ICP, particularly in patients with reduced intracranial compliance [5]. These issues highlight the need for refined strategies that safeguard both pulmonary and cerebral function [2].

The increasing use of advanced neuromonitoring—including ICP monitoring, brain tissue oxygenation, transcranial Doppler, and esophageal pressure—has enabled clinicians to assess, in real time, the cerebral impact of ventilatory settings [6]. This multimodal approach has enhanced clinical decision-making and underscored the bidirectional interplay between the lungs and brain [7]. Moreover, pulmonary complications such as ventilator-associated pneumonia (VAP), ARDS, and neurogenic pulmonary edema (NPE) are now recognized as integral to the trajectory of brain injury [8]. Recently, adjunctive therapies such as prone positioning and extracorporeal membrane oxygenation (ECMO) have gained attention for managing severe respiratory failure in brain-injured patients, although these interventions may further challenge cerebral homeostasis [9].

**Fig. 1:**
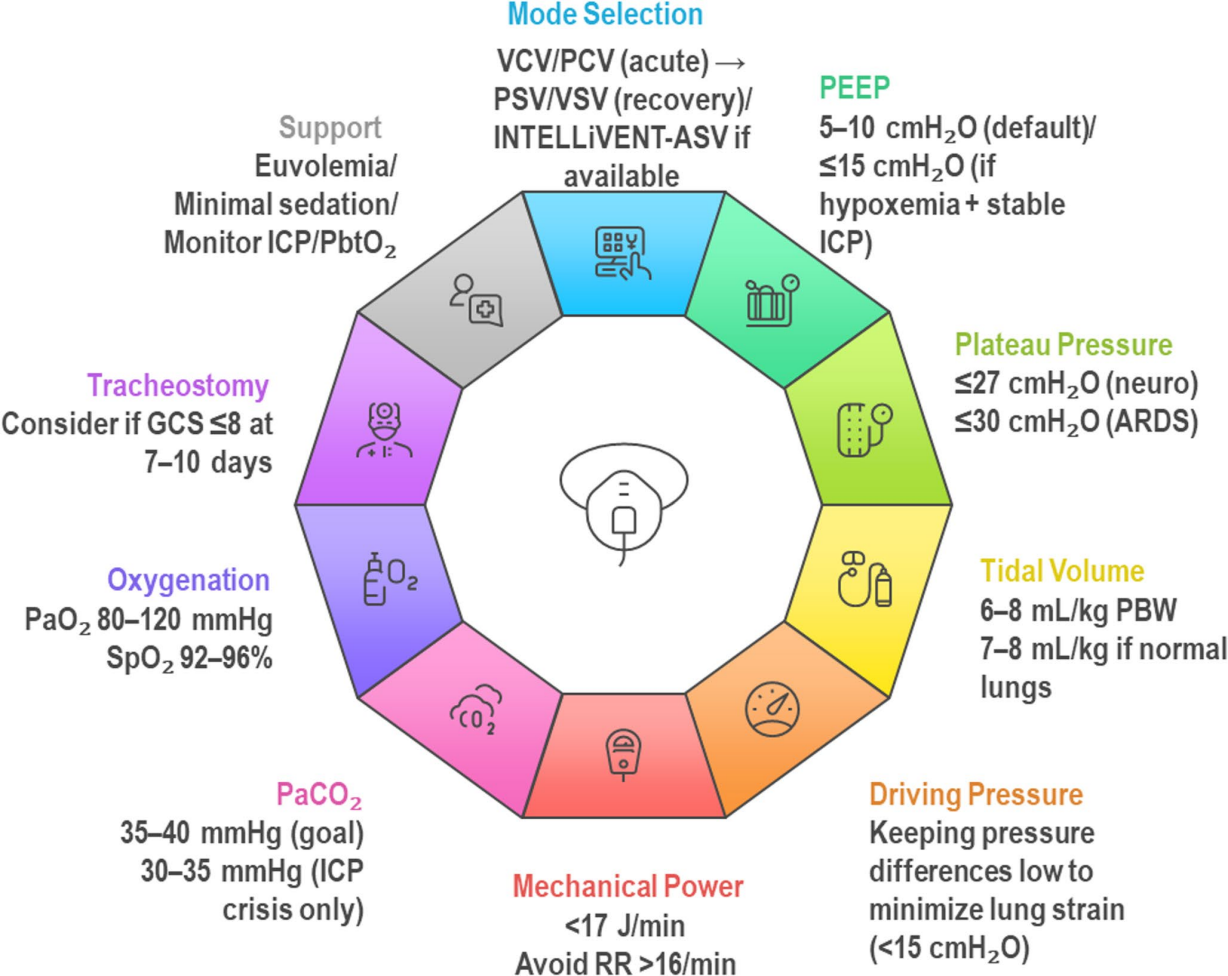
Mechanical ventilation in acute brain injury requires a carefully tailored approach that balances lung protection with cerebral preservation. The following ten principles integrate respiratory and neurophysiological goals to minimize secondary brain injury: 1. Ventilator Mode Selection: Use VCV or PCV in the acute phase to ensure controlled ventilation. Transition to assisted modes (PSV, VSV) as neurological and respiratory stability improve, with close monitoring for ICP fluctuations. 2. Positive End-Expiratory Pressure (PEEP): Apply moderate PEEP (5–10 cm H₂O) to maintain alveolar recruitment and oxygenation. Titrate cautiously with ICP monitoring to avoid impairing cerebral venous return. 3. Plateau Pressure (Pplat): Maintain Pplat ≤27–30 cm H₂O to minimize ventilator-induced lung injury (VILI) and prevent secondary cerebral insults due to impaired venous drainage or increased intrathoracic pressure. 4. Tidal Volume (VT): Adjust VT to 6–8 mL/kg PBW, guided by Pplat and ICP. Moderate volumes (7–8 mL/kg) may support PaCO₂ control in patients with preserved compliance, while lower VT may be necessary in ARDS. 5. Driving Pressure (ΔP): Target ΔP <15 cm H₂O to reduce lung stress and systemic inflammation that may exacerbate cerebral edema and disrupt the blood-brain barrier. 6. Mechanical Power (MP): Limit MP to <17 J/min to minimize cumulative energy delivery and ventilator-associated injury. Avoid excessive RR or VT that disproportionately raise MP, particularly in patients with low compliance. 7. PaCO₂ Targets: Maintain normocapnia (PaCO₂ 35–40 mmHg). Use short-term hypocapnia (30–35 mmHg) during acute ICP crises with caution. Continuous end-tidal CO₂ and arterial blood gas monitoring are essential. 8. Oxygenation Goals: Avoid both hypoxemia (PaO₂ <60 mmHg) and hyperoxemia (PaO₂ >120–150 mmHg). Aim for PaO₂ 80–120 mmHg (SpO₂ 92–96%) and consider PbtO₂ monitoring (target 20–30 mmHg) for individualized management. 9. Tracheostomy Timing: Do not rely on rigid timing protocols. Base the decision on neurological trajectory, sedation needs, and predicted duration of ventilation. Early tracheostomy may facilitate weaning and neuroassessment in selected patients. 10. Supportive Strategies: Integrate euvolemia, light sedation, and multimodal neuromonitoring (ICP, PbtO₂, autoregulation indices) into ventilation management. These elements guide safe ventilator titration and reduce secondary brain injury risk. *Abbreviations*: VCV = Volume-Controlled Ventilation; PCV = Pressure-Controlled Ventilation; PSV = Pressure Support Ventilation; VSV = Volume Support Ventilation; GCS = Glasgow Coma Scale; ICP = Intracranial Pressure; PbtO₂ = Brain Tissue Oxygenation; PaO₂ = Partial Pressure of Arterial Oxygen; SpO₂ = Peripheral Oxygen Saturation; PaCO₂ = Partial Pressure of Arterial Carbon Dioxide; PEEP = Positive End-Expiratory Pressure; ARDS = Acute Respiratory Distress Syndrome; PBW = Predicted Body Weight; RR = Respiratory Rate

This review synthesizes ten evidence-based principles of mechanical ventilation in acute brain injury, offering a practical framework for clinicians to balance cerebral protection with lung-protective strategies. Conditions addressed include TBI, stroke, subarachnoid hemorrhage, and hypoxic–ischemic encephalopathy. These principles are summarized in Fig. 1 and focus on optimizing tidal volume (VT), PEEP, oxygenation targets, and integration of neuromonitoring to minimize secondary injury while supporting pulmonary function.

## Physiological Goals Should Guide Ventilator Mode Selection

In patients with acute brain injury (ABI), the choice of ventilator mode should be guided by physiological goals—particularly the need for stable cerebral perfusion and precise control of arterial carbon dioxide (PaCO₂) levels [10, 11]. Volume-controlled ventilation (VCV) remains the most commonly used mode due to its ability to deliver predictable VTs and facilitate consistent PaCO₂ control [12]. Pressure-controlled ventilation (PCV), including pressure-control assist/control (PC-AC), may be preferable in patients with reduced lung compliance or elevated risk of barotrauma [13].

Among closed-loop ventilation systems, INTELLiVENT-ASV is currently the only mode with demonstrated potential to optimize ventilation in ABI patients [14]. It automatically adjusts VT, respiratory rate, and PEEP on a breath-by-breath basis to target specific end-tidal CO₂ and SpO₂ values, guided by real-time assessments of gas exchange and lung mechanics. In high-risk neurocritical care settings—where continuous manual titration is often unfeasible—this technology may support individualized, precise ventilatory management essential for brain protection [14].

Maintaining normocapnia is crucial in ABI, as both hypo- and hypercapnia are associated with secondary brain injury. Hypocapnia can reduce cerebral blood flow and exacerbate ischemia, while hypercapnia may increase intracranial pressure (ICP), particularly in patients with impaired cerebral autoregulation. Therefore, ventilator settings should aim to maintain PaCO₂ within the normal range, with frequent monitoring via arterial blood gases or continuous end-tidal CO₂, when available [14–16].

As patients stabilize, transitioning to assisted ventilation modes may facilitate weaning and reduce sedation requirements. Options such as pressure support ventilation (PSV) or volume support ventilation (VSV) can be considered, but require close monitoring to avoid over-assistance and prevent fluctuations in PaCO₂ that could destabilize ICP [17, 18]. In patients with improving consciousness or spontaneous breathing, assisted modes may enhance comfort and reduce time to extubation. However, they should be implemented cautiously and ideally in conjunction with neuromonitoring to ensure cerebral stability [17, 18].

In summary, PCV or VCV are preferred during the acute phase of ABI, particularly when deep sedation is required. Transition to assisted modes should occur as soon as neurological and respiratory conditions permit, always under close physiological monitoring. Ventilator mode selection should be tailored to the individual’s clinical trajectory rather than applied according to rigid protocols.

## PEEP Can Be Safely Used with Cerebral Monitoring

PEEP is essential for maintaining alveolar recruitment, improving oxygenation, and preventing atelectasis in mechanically ventilated patients [5, 19]. In individuals with ABI, its application requires caution due to potential increases in intrathoracic pressure and ICP [5, 20]. However, moderate PEEP levels (5–10 cm H₂O) are generally well tolerated, provided that hemodynamic stability is preserved [11].

Although PEEP may theoretically reduce cerebral venous return and thereby increase ICP, its actual effect is highly variable and context-dependent. Factors such as intravascular volume status, baseline lung and chest wall compliance, degree of lung recruitability, and underlying cerebral compliance all influence the net effect of PEEP on intracranial dynamics [20].

When cerebral compliance is intact and intrathoracic pressures are optimized, PEEP can often be titrated upward to enhance oxygenation without compromising cerebral physiology [5, 20]. Importantly, PEEP should be applied in a goal-directed manner, aiming to optimize arterial oxygenation (PaO₂) while vigilantly monitoring for potential cerebral side effects [11]. Invasive ICP monitoring remains the gold standard for evaluating PEEP tolerance [6]. However, in its absence, clinicians must rely on indirect clinical signs such as hypertension, bradycardia, altered mental status, and pupillary changes [10, 21].

PEEP becomes particularly critical when ABI patients develop pulmonary complications such as ARDS or neurogenic pulmonary edema [8, 22]. In these scenarios, lung-protective strategies—including appropriate PEEP—should not be withheld solely due to concerns about ICP [5, 11]. Indeed, hypoxemia, atelectasis, and impaired oxygen delivery may pose greater risks to cerebral tissue [5]. Supportive measures such as head-of-bed elevation, normovolemia, and adequate sedation [23, 24], can mitigate potential adverse effects and enhance PEEP tolerance [5, 20].

Current evidence suggests that PEEP is not contraindicated in neurocritical care, but should be applied with a physiology-informed, context-aware approach. As in general ICU practice, PEEP should be tailored to individual lung mechanics and gas exchange goals, while incorporating cerebral monitoring to prevent secondary brain injury.

The optimal PEEP level should be determined individually, rather than by fixed thresholds. While lower PEEP values are typically preferred to maintain CPP, particularly when kept below concurrent ICP values [11, 20], higher PEEP (up to 15 cm H₂O) may be cautiously applied in hemodynamically stable, euvolemic patients, provided that rigorous monitoring confirms stable ICP and adequate CPP [5, 11, 13, 24].

Tolerance to higher PEEP should be assessed through continuous evaluation of ICP reactivity, cerebral oxygenation, and systemic hemodynamics following each adjustment [11, 20]. This physiology-driven strategy prioritizes cerebral perfusion, while allowing for individualized alveolar recruitment when the potential pulmonary benefits outweigh cerebral risks [5, 11].

Head-of-bed elevation to at least 30 degrees should accompany PEEP titration to promote venous drainage and mitigate ICP elevation [20, 24]. Exceeding conventional PEEP levels should only be considered when refractory hypoxemia persists despite lower settings and should be guided by arterial blood gas analysis and real-time neuromonitoring data [5, 11, 13].

## Protective Plateau Pressures Should be Maintained

Pplat, measured during an end-inspiratory pause, reflects alveolar and small airway pressure and serves as a surrogate marker of lung stress and static compliance. Maintaining Pplat within a protective range is associated with reduced VILI, a critical consideration in patients with ABI, who often require prolonged mechanical ventilation.

In patients with ARDS, clinical guidelines recommend limiting Pplat to ≤ 30 cmH₂O to reduce the risk of VILI [19, 25]. However, stricter thresholds may be warranted in neurocritical care. The PROLABI trial showed that ABI patients without ARDS may benefit from keeping Pplat below 25 cmH₂O, as higher values were associated with worse neurological outcomes [26]. Supporting this, the VENTIBRAIN international observational study found that higher Pplat was independently associated with increased ICU and 6-month mortality in ABI patients (hazard ratio: 1.50; 95% CI: 1.27–1.78), although its relationship with neurological outcomes was less robust [27]. These findings align with evidence from ARDS research suggesting that driving pressure (ΔP = Pplat − PEEP) may be an even better predictor of mortality than Pplat alone [28], suggesting the need to tailor respiratory mechanics to both pulmonary and cerebral physiology.

In patients with isolated brain injury and preserved lung compliance, low VTs (6 mL/kg predicted body weight) are usually sufficient to maintain safe Pplat levels [10, 11, 26]. However, when lung compliance is impaired—such as in ARDS [19, 25], ventilator-associated pneumonia [8], or neurogenic pulmonary edema [8, 22], maintaining a protective Pplat can be challenging, even with reduced VTs [13, 28].

Elevated Pplat is not only harmful to the lungs but may also exacerbate secondary brain injury by impairing gas exchange and promoting systemic inflammation [29]. Overdistension and increased intrathoracic pressure can lead to cytokine release, disruption of the alveolar-capillary barrier, and breakdown of the blood–brain barrier, contributing to cerebral edema [8, 13]. In addition, high airway pressures may impede cerebral venous outflow, potentially increasing ICP and compromising cerebral perfusion.

Routine monitoring of Pplat is essential, particularly after ventilator adjustments or clinical deterioration [11, 28]. If Pplat exceeds 30 cmH₂O, clinicians should consider reducing VT, adjusting PEEP, or optimizing the inspiratory flow pattern [19, 28]. These interventions must be balanced against real-time monitoring of ICP and CPP to maintain cerebral homeostasis [6, 10].

We recommend maintaining Pplat ≤ 27 cmH₂O in brain-injured patients to minimize VILI and support cerebral protection. This strategy should be integrated with regular assessments of static compliance and ICP trends, especially in patients with reduced lung compliance due to ARDS or neurogenic pulmonary edema. In such patients, more frequent monitoring and ventilator adjustments may be necessary to preserve both pulmonary integrity and cerebral perfusion.

## Tidal Volumes Should be Adjusted Within Safe Pressure Limits

The low VT strategy (6 mL/kg predicted body weight) is a well-established cornerstone of lung-protective ventilation in both ARDS and non-ARDS populations [19]. Nevertheless, its application in patients with ABI is complex, given the need to balance pulmonary protection with the risks of hypercapnia-induced elevations in ICP [4, 10]. Conversely, excessive VTs may exacerbate VILI. Emerging data from the PROLABI trial suggest that moderate VTs (7–8 mL/kg) may offer a favorable compromise in ABI patients with preserved lung compliance and Pplat ≤ 30 cmH₂O [26]. This strategy demands close monitoring of ΔP, PaCO₂, and ICP to avoid secondary cerebral or pulmonary injury.

In patients with ABI but intact lung mechanics, moderate VTs (up to 7–8 mL/kg) may support adequate CO₂ clearance while maintaining safe Pplat and ΔP [10, 11]. This is particularly relevant in cases where strict low-volume ventilation may provoke hypercapnia and cerebral vasodilation, thereby increasing ICP [5, 10]. A multicenter study demonstrated that flexibility in tidal volume, within protective pressure thresholds, did not worsen pulmonary outcomes and could facilitate normocapnia in neurocritical care settings [11].

However, caution is warranted. Even modest increases in tidal volume may raise Pplat and ΔP, especially in patients with subclinical lung injury or evolving ARDS, necessitating ongoing reassessment of lung mechanics [4, 5]. The ARDSNet trial [19] showed that low VT (6 mL/kg) significantly reduced mortality compared to traditional settings (VT = 12 mL/kg), underscoring the dangers of overdistension in injured lungs. While the Berlin Definition [25] provides a framework for ARDS severity classification, it does not specify ventilatory adjustments; subsequent guidelines emphasize the importance of monitoring Pplat and ΔP to avoid volutrauma.

In ABI patients, even subtle ventilator-induced changes can influence cerebral physiology, including ICP plateau waves and hemodynamic instability [30]. While the ARDSNet strategy [19] remains a safe default for patients with compromised lung compliance, recent meta-analyses suggest that low tidal volumes with moderate PEEP do not worsen outcomes in ABI and may improve oxygenation [10]. Thus, ventilatory strategies in ABI should be personalized, incorporating real-time neuromonitoring, such as ICP waveform analysis [6, 30], to guide safe and effective care.

We recommend individualizing VTs within the 6–8 mL/kg range, prioritizing maintenance of Pplat < 30 cmH₂O, protective ΔP, and stable ICP trends. In patients with preserved lung compliance, moderate tidal volumes (7–8 mL/kg) may enhance CO₂ clearance without compromising cerebral or pulmonary safety. All tidal volume adjustments should be followed by reassessment of Pplat, PaCO₂, and ICP, ensuring that both pulmonary mechanics and cerebral hemodynamics remain within acceptable ranges.

## Driving Pressure Should be Minimized to Reduce Lung and Brain Injury

ΔP reflects the cyclic stress and strain imposed on alveoli during mechanical ventilation. A landmark study [28] demonstrated that ΔP is the strongest independent predictor of mortality in ARDS, with each 7 cm H₂O increase in ΔP associated with a 41% rise in mortality, regardless of tidal volume or PEEP settings [28, 31]. These findings underscore ΔP as a pivotal determinant of VILI.

In the neurocritical care setting, elevated ΔP carries dual physiological risks: it not only exacerbates lung injury but also triggers systemic inflammatory responses that may worsen cerebral edema and disrupt the blood–brain barrier (BBB) [8]. Preclinical models, including blast lung injury (BLI) experiments [29], demonstrate that higher ΔP is associated with pulmonary hemorrhage, alveolar inflammation, and impaired gas exchange—manifested as elevated CO₂ gap and alveolar–arterial oxygen gradients (A–aDO₂)—all of which may propagate secondary brain injury. Clinically, pulmonary complications such as ARDS and neurogenic pulmonary edema, which occur in 20–30% of severe TBI cases [22], are independently associated with worse neurological outcomes.

Accordingly, ΔP-targeted ventilation strategies—aiming to maintain ΔP < 15 cm H₂O—have become central to both pulmonary protection and neuroprotection [28]. While low VT (6 mL/kg) remain fundamental in brain injury patients [10], optimizing ΔP may be more effective in minimizing both pulmonary and neurological sequelae [8, 22, 28]. A post hoc analysis of the EPVent2 trial reinforced ΔP as a superior predictor of outcomes—even when Pplat or VT alone appear within acceptable ranges [31]. Therefore, in brain-injured patients, especially those with ARDS, pneumonia, or neurogenic pulmonary edema, ΔP should be routinely monitored and prioritized during ventilator titration.

Reducing ΔP may involve a combination of strategies: (1) Reducing tidal volume, where feasible; (2) Optimizing PEEP to improve respiratory system compliance; (3) Minimizing patient–ventilator asynchrony through adequate sedation and ventilator mode adjustment; and, 4)where available, using esophageal pressure monitoring to estimate transpulmonary ΔP and guide individualized PEEP titration [28, 29, 31, 32]. Importantly, elevated ΔP can be present even when Pplat remains ≤ 30 cmH₂O, highlighting the need for ΔP-specific monitoring to detect “hidden” mechanical stress [28, 31].

In the context of ABI, ΔP minimization is not merely a lung-protective measure—it is a neuroprotective strategy. Pulmonary-driven inflammation, impaired oxygenation, and hemodynamic shifts directly impact cerebral homeostasis. Therefore, ΔP represents a critical interface between lung and brain physiology. Keeping ΔP low aligns with the broader goals of multi-organ protective ventilation [5, 13, 32].

We recommend maintaining ΔP ≤ 15 cm H₂O as a core component of mechanical ventilation in ABI. When feasible, optimize PEEP first to recruit lung units and reduce ΔP before lowering VT, thus preserving oxygenation while minimizing lung strain. Clinicians should integrate ΔP with measurements of Pplat, systemic oxygenation, and cerebral oxygenation (e.g., brain tissue oxygen tension, PbtO₂) to guide ventilatory strategies that are safe for both lungs and brain.

## Mechanical Power and Composite Parameters Require Close Monitoring

Mechanical power (MP) represents the total energy delivered to the respiratory system per unit of time and incorporates multiple ventilatory variables, such as VT, ΔP, RR, and PEEP [33]. MP has emerged as a comprehensive predictor of VILI, as it reflects the cumulative mechanical load imposed on lung tissue over time [34]. In patients with ABI, excessive MP may worsen systemic inflammation, disrupt the blood–brain barrier, and contribute to cerebral edema, reinforcing the need for careful monitoring and titration [8, 13].

MP becomes particularly relevant in patients with ARDS or decreased lung compliance, where small increases in RR or VT can substantially elevate energy transfer to the lungs [35]. This is especially problematic in neurocritical care, where RR is often increased to reduce PaCO₂ and manage ICP. However, such adjustments may inadvertently elevate MP and intensify VILI risk [36]. A simplified formula (e.g., MP ≈ 0.098 × VT × RR × [Ppeak − 0.5 × ΔP]) can be used for bedside estimation [33], but clinical emphasis should remain on balancing cerebral and pulmonary goals [34]. Strategies to limit MP include: (1) maintaining low to moderate VTs (6–8 mL/kg PBW); (2) avoiding excessive RR, especially in normocapnic or marginally hypocapnic patients; and (3) optimizing PEEP to improve compliance and reduce ΔP [34, 35].

Importantly, MP offers additional insight beyond traditional metrics such as Pplat and ΔP. For instance, a patient may have an acceptable Pplat yet still be exposed to high MP due to elevated RR or inspiratory flow [37]. As such, MP serves as a valuable composite parameter for detecting “hidden” ventilatory stress, particularly in brain-injured patients receiving prolonged mechanical ventilation.

Emerging data supports the integration of MP into ventilatory management protocols for ABI, as part of a broader multi-organ protective strategy [33, 36]. The cumulative mechanical burden imposed by ventilation not only increases pulmonary risk but may also amplify neuroinflammation and compromise cerebral autoregulation [13].

We recommend targeting MP < 17 J/min in patients requiring prolonged ventilation to minimize VILI risk. Particular attention should be given to avoiding disproportionately high RR, as it contributes more to MP than adjustments in PEEP or VT. Whenever possible, monitoring MP alongside inflammatory biomarkers (e.g., plasma IL-6 or SP-D) and neuromonitoring data (e.g., ICP, PbtO₂) may help tailor ventilation strategies that are protective for both lungs and brain.

## PaCO₂ Should be Titrated Based on ICP and Cerebral Blood Flow

PaCO₂ is a critical modulator of CBF and ICP in in patients with ABI [4, 10]. Hypercapnia induces cerebral vasodilation, increasing CBF and ICP, while hypocapnia causes vasoconstriction, reducing both [21]. Historically, therapeutic hyperventilation was employed to lower ICP, but prolonged hypocapnia may precipitate cerebral ischemia, particularly in regions with impaired autoregulation [21].

Current guidelines recommend maintaining normocapnia (PaCO₂ 35–40 mmHg) as the default target in ABI [10, 21]. However, individualized adjustments based on real-time monitoring are often warranted [10, 21]. For example, mild permissive hypercapnia (PaCO₂ ~45 mmHg) may be acceptable in patients with stable ICP and low PbtO₂, potentially enhancing cerebral oxygen delivery [38]. Transient hypocapnia (PaCO₂ 30–35 mmHg) may be used during acute ICP elevations but should be reversed promptly to minimize the risk of cerebral ischemia [39].

Multimodal neuromonitoring—including ICP catheters, PbtO₂ probes, and transcranial Doppler (TCD)—provides essential guidance for PaCO₂ titration [6, 30]. In settings without advanced monitoring, careful tracking of ICP trends, neurological status, and arterial blood gases (ABG) remains critical [10].

In short, normocapnia (PaCO₂ 35–40 mmHg) should remain the standard target in ABI. Transient hypocapnia (30–35 mmHg) may be used for acute ICP control but should be discontinued as soon as ICP stabilizes. Continuous end-tidal CO₂ (EtCO₂) monitoring, paired with hourly ABG correlation, is essential to guide safe and precise PaCO₂ management, balancing CBF and ICP.

## Hypoxemia and Hyperoxemia Should both be Avoided

Optimal oxygenation is fundamental in neurocritical care, as both hypoxemia (PaO₂ <60 mmHg) and hyperoxemia (PaO₂ >120–150 mmHg) are associated with worse outcomes in ABI [40]. Hypoxemia compromises cerebral oxygen delivery, exacerbating neuronal injury, while hyperoxemia may promote oxidative stress, disruption of the blood-brain barrier, and cerebral vasoconstriction, leading to secondary injury [41].

A U-shaped relationship between PaO₂ and mortality has been reported, with both extremes increasing the risk of adverse outcomes [42]. Current evidence supports targeting a PaO₂ of 80–120 mmHg (corresponding to SpO₂ 92–96%), using the lowest FiO₂ necessary to maintain this range [40]. Importantly, prolonged exposure to 100% FiO₂ should be avoided to reduce the risk of oxygen toxicity [41].

PbtO₂ monitoring adds valuable granularity in patients with compromised autoregulation. If PbtO₂ remains low despite adequate systemic oxygenation, the clinical focus should shift to enhancing cerebral perfusion (e.g., optimizing CPP), rather than escalating FiO₂ [42, 43]. The ENIO study and subsequent analyses emphasize the importance of individualized oxygenation thresholds, guided by multimodal neuromonitoring and assessment of cerebral autoregulation [44].

In summary, maintain PaO₂ between 80 and 120 mmHg (SpO₂ 92–96%) as a general target. Avoid sustained use of FiO₂ 1.0. When available, incorporate PbtO₂ monitoring (target 20–30 mmHg) and autoregulation assessments to tailor oxygenation strategies. In selected patients, individualized thresholds, informed by neuromonitoring, may offer superior protection against both hypoxic and hyperoxic injury.

## Tracheostomy Timing Should be Individualized in Brain-Injured Patients

The optimal timing of tracheostomy in patients with acute brain injury remains a complex and nuanced clinical decision. Recent evidence from a large, multicenter UK trial found no significant difference in clinical outcomes between early tracheostomy (within 4 days of initiation of mechanical ventilation) and late tracheostomy (after 10 days, if still required). Thirty-day mortality rates were nearly identical (30.8% vs. 31.5%), as were two-year survival rates (51.0% vs. 53.7%), ICU length of stay (13.0 vs. 13.1 days), and complication rates (5.5% vs. 7.8%). These findings challenge the presumption that early tracheostomy confers universal benefits in critically ill populations.

Importantly, the trial also revealed the limited ability of clinicians to predict which patients would require prolonged mechanical ventilation, underscoring the need for a more dynamic, patient-specific approach. In the context of acute brain injury, the decision to perform tracheostomy should be guided by the patient’s neurological trajectory, level of consciousness, expected recovery, and evolving respiratory needs, rather than by rigid time-based protocols [45].

Although early tracheostomy does not appear to improve survival or reduce ICU stay across unselected cohorts, it may offer specific benefits in neurocritical care populations, including: (1) reduced sedation requirements, facilitating more frequent and accurate neurological assessments, (2) enhanced patient comfort and reduced risk of ventilator-induced delirium, (3) Improved secretion clearance and reduced work of breathing, especially in patients with bulbar dysfunction or impaired cough reflex [46], and Lower rates of ventilator-associated pneumonia (VAP) and possibly shortened duration of mechanical ventilation, particularly when performed in the early phase of illness [39].

However, these benefits must be weighed against procedure-related risks, such as bleeding, infection, and tracheal stenosis. While percutaneous tracheostomy is increasingly favored for its bedside feasibility, it may carry a higher risk of early procedural complications compared to the surgical approach, though long-term outcomes are similar.

However, these benefits must be weighed against procedure-related risks, such as bleeding, infection, and tracheal stenosis. While percutaneous tracheostomy is increasingly favored for its bedside feasibility, it may carry a higher risk of early procedural complications compared to the surgical approach, though long-term outcomes are similar [47]. In contrast, those showing signs of neurological improvement or with reversible causes of respiratory failure may safely defer or avoid the procedure [48].

Ultimately, the timing of tracheostomy in ABI should be individualized [49, 50], supported by: (1) multidisciplinary discussions involving intensivists, neurologists, respiratory therapists, and when possible, neurosurgeons, (2) Engagement of family members or surrogate decision-makers, ensuring alignment with patient values and goals of care [51], and (3) utilization of emerging predictive tools, including clinical and radiological criteria, to guide decision-making in the absence of absolute indicators [47].

In summary, tracheostomy should not be performed routinely or based solely on ventilator day counts. Instead, it should be tailored to the individual patient’s clinical progression, particularly their neurological status and anticipated duration of mechanical ventilation. In selected cases, early tracheostomy may facilitate weaning, improve comfort, and support more effective neuro-monitoring. Future research should focus on refining prediction models to better identify patients most likely to benefit from early intervention.

## Fluid Balance, Sedation, and Neuromonitoring are Essential for Ventilation Success

Optimal mechanical ventilation in brain-injured patients requires precise integration of sedation, fluid management, and neuromonitoring, as these factors collectively influence both respiratory mechanics and cerebral physiology. Misalignment in any of these domains can exacerbate secondary brain injury and compromise outcomes [52].

Sedation serves a dual role in neurocritical care. In the acute phase, it facilitates ventilator synchrony, reduces metabolic demand, and suppresses deleterious spikes in ICP caused by coughing or agitation [53]. However, excessive or prolonged sedation can obscure neurological deterioration, delay weaning, and contribute to ICU-acquired weakness [54].

Current guidelines advocate for the lowest effective sedative dose, with daily sedation interruption whenever clinically feasible to enable neurological evaluation [23]. Agent selection should consider cerebral effects; for instance, propofol is often favored over midazolam due to its shorter half-life and beneficial effects on cerebral metabolic rate and ICP [24].

Fluid management presents unique challenges in ventilated neurocritical patients. Hypovolemia can compromise CPP, particularly in patients with impaired autoregulation, while fluid overload may worsen pulmonary edema and gas exchange [11].

Best practices emphasize the maintenance of euvolemia, typically with balanced crystalloids, guided by daily weights, fluid input/output, and when available, advanced hemodynamic monitoring [55]. In patients with coexisting ARDS, the need to limit extravascular lung water must be carefully balanced with preserving cerebral perfusion [11].

Advanced neuromonitoring provides real-time insight into the cerebral consequences of ventilatory adjustments. Continuous ICP monitoring is indispensable for assessing tolerance to changes in VT, PEEP, or PaCO₂ [6]. PbtO₂ offers direct assessment of cerebral oxygen delivery and may refine decisions regarding oxygenation and ventilation targets [43]. Multimodal monitoring, including indices of cerebral autoregulation (e.g., pressure reactivity index, PRx), allows for titration of CPP toward individualized “optimal” targets, aligning hemodynamic and ventilatory goals [56].

The integration of these components - sedation, fluids, and neuromonitoring - an interdependent feedback system that should guide ventilator management: adjustments in PEEP or VT should be accompanied by simultaneous evaluation of ICP, CPP, and PbtO₂ [57]. Similarly, fluid boluses or diuretic therapy must be evaluated for their impact on both cerebral perfusion and pulmonary function [58]. This system-based, multimodal strategy reflects the evolving standard in neurocritical care, where ventilation cannot be managed in isolation but must be coordinated with comprehensive physiological support [59].

In short, use protocolized sedation interruption, guided by multimodal neuromonitoring, to optimize ventilation while preserving neurological assessment. Maintain euvolemia through targeted fluid strategies to protect both cerebral and pulmonary function. Continuous tracking of ICP, CPP, PbtO₂, and fluid balance is essential to tailor ventilation and supportive care in brain-injured patients.

## Conclusion

Mechanical ventilation in acute brain injury requires a delicate balance between protecting pulmonary function and preserving cerebral physiology. The principles discussed underscore the importance of individualized ventilator strategies that prioritize physiological stability over protocol-driven approaches. Clinicians should aim to maintain effective gas exchange while carefully monitoring the cerebral consequences of ventilatory interventions.

Protective ventilation—anchored in controlled airway pressures, personalized tidal volumes, and judicious application of PEEP—helps minimize VILI without compromising cerebral perfusion. Meticulous control of PaCO₂ and avoidance of both hypoxemia and hyperoxemia are critical to preventing secondary brain injury. Integration of advanced neuromonitoring tools, including ICP, PbtO₂, and cerebral autoregulation indices, enables real-time titration of ventilatory parameters to meet both pulmonary and neurological goals.

The timing of tracheostomy should be guided by the individual patient’s neurological trajectory and anticipated duration of mechanical ventilation, rather than fixed timelines. Similarly, fluid balance and sedation strategies must be optimized to support respiratory function while preserving cerebral perfusion and enabling accurate neurological assessment.

Looking ahead, future research should focus on refining ventilation protocols to enhance long-term neurological recovery and further elucidate the interplay between lung and brain physiology. Among emerging technologies, INTELLiVENT-ASV represents a significant advancement in neurocritical care. By automating real-time adjustments to tidal volume, respiratory rate, and PEEP based on end-tidal CO₂ and SpO₂ targets, and integrating with multimodal neuromonitoring (e.g., ICP, PbtO₂, cerebral autoregulation indices), it offers a validated, precision-based approach to brain-lung management—surpassing conventional modes in safety and individualization for high-risk patients.

By thoughtfully applying these physiology-guided strategies, clinicians can more effectively safeguard both pulmonary and neurological function, ultimately improving outcomes for patients with ABI.

## Key References

- Asehnoune K, Rooze P, Robba C, Bouras M, Mascia L, Cinotti R, et al. Mechanical ventilation in patients with acute brain injury: a systematic review with meta-analysis. Crit Care. 2023;27(1):221.
  - Meta-analysis exploring the role of lung protective ventilation in acute brain injured patients.
- Robba C, Poole D, McNett M, Asehnoune K, Bösel J, Bruder N, et al. Mechanical ventilation in patients with acute brain injury: recommendations of the European Society of Intensive Care Medicine consensus. Intensive Care Med. 2020;46(12):2397-410.
  - Most recent Guidelines for the management of mechanically ventilated brain injured patients.
- Robba C, Giardiello D, Almondo C, Asehnoune K, Badenes R, Cinotti R, et al. Ventilation practices in acute brain injured patients and association with outcomes: the VENTIBRAIN multicenter observational study. Intensive Care Med. 2025;51(2):318-31.
  - Largest observational study on mechanical ventilation in acute brain injured patients.

## Data Availability

No datasets were generated or analysed during the current study.
